# Genetic Ablation of Type III Adenylyl Cyclase Exerts Region-Specific Effects on Cilia Architecture in the Mouse Nose

**DOI:** 10.1371/journal.pone.0150638

**Published:** 2016-03-04

**Authors:** Rosemary C. Challis, Huikai Tian, Wenbin Yin, Minghong Ma

**Affiliations:** 1 Department of Neuroscience, University of Pennsylvania Perelman School of Medicine, Philadelphia, PA 19104, United States of America; 2 Department of Geriatrics, Qilu Hospital of Shandong University, Jinan, Shandong 250012, China; The University of Tokyo, JAPAN

## Abstract

We recently reported that olfactory sensory neurons in the dorsal zone of the mouse olfactory epithelium exhibit drastic location-dependent differences in cilia length. Furthermore, genetic ablation of type III adenylyl cyclase (ACIII), a key olfactory signaling protein and ubiquitous marker for primary cilia, disrupts the cilia length pattern and results in considerably shorter cilia, independent of odor-induced activity. Given the significant impact of ACIII on cilia length in the dorsal zone, we sought to further investigate the relationship between cilia length and ACIII level in various regions throughout the mouse olfactory epithelium. We employed whole-mount immunohistochemical staining to examine olfactory cilia morphology in phosphodiesterase (PDE) 1C^-/-^;PDE4A^-/-^ (simplified as PDEs^-/-^ hereafter) and ACIII^-/-^ mice in which ACIII levels are reduced and ablated, respectively. As expected, PDEs^-/-^ animals exhibit dramatically shorter cilia in the dorsal zone (i.e., where the cilia pattern is found), similar to our previous observation in ACIII^-/-^ mice. Remarkably, in a region not included in our previous study, ACIII^-/-^ animals (but not PDEs^-/-^ mice) have dramatically elongated, comet-shaped cilia, as opposed to characteristic star-shaped olfactory cilia. Here, we reveal that genetic ablation of ACIII has drastic, location-dependent effects on cilia architecture in the mouse nose. These results add a new dimension to our current understanding of olfactory cilia structure and regional organization of the olfactory epithelium. Together, these findings have significant implications for both cilia and sensory biology.

## Introduction

Cilia are remarkably diverse in their numbers, lengths, and morphologies and are therefore well-suited for mediating various cellular functions, such as fluid transport, cell motility, and detection of sensory stimuli [1, 2]. Defects in cilia structure and/or function cause a host of diseases, which can manifest in multiple organs and result in severe sensory impairments like anosmia, or loss of smell [3–5]. Understanding how cilia architecture (i.e., length and/or shape) and function are regulated in different cell types is critical for the future therapeutic treatment of these disorders.

The mammalian olfactory system is a unique model for studying cilia structure and function, given that olfactory sensory neurons (OSNs) in the mouse nose possess cilia with different lengths and sensitivities, depending on the cell location in the olfactory epithelium [6]. Each OSN can possess up to 30 immotile cilia, which extend in a star-like arrangement from the dendritic knob and house the key proteins involved in olfactory signal transduction [7, 8]. Odor detection begins when odor molecules bind to odorant receptors on the ciliary membrane, leading to G protein (G_olf_)-dependent activation of type III adenylyl cyclase (ACIII) and an increase in intraciliary cyclic adenosine monophosphate (cAMP) levels. Opening of downstream channels, including a cyclic nucleotide-gated channel, depolarizes OSNs, which relay action potentials to the brain for odor processing [9, 10]. Termination and adaptation of the olfactory response is critical for proper olfactory signaling and is mediated in part by phosphodiesterases (PDEs), which hydrolyze cAMP to AMP [11].

Curiously, several studies have reported a role of olfactory signaling molecules in shaping olfactory cilia architecture in both *Caenorhabditis elegans* and mice [6, 12–14]. These data suggest that signaling components may play dual roles in sensing external stimuli and sculpting the shape of a cilium. We previously reported that ACIII, a key olfactory signaling molecule and ubiquitous marker for primary cilia [15], is required for mediating olfactory cilia length and the establishment of the cilia pattern in the dorsal zone of the mouse olfactory epithelium [6]. Here, we further explore the relationship between cilia structure and ACIII level in various locations throughout the mouse nose.

## Results

### Genetic ablation of PDEs disrupts the cilia pattern

**Fig 1:**
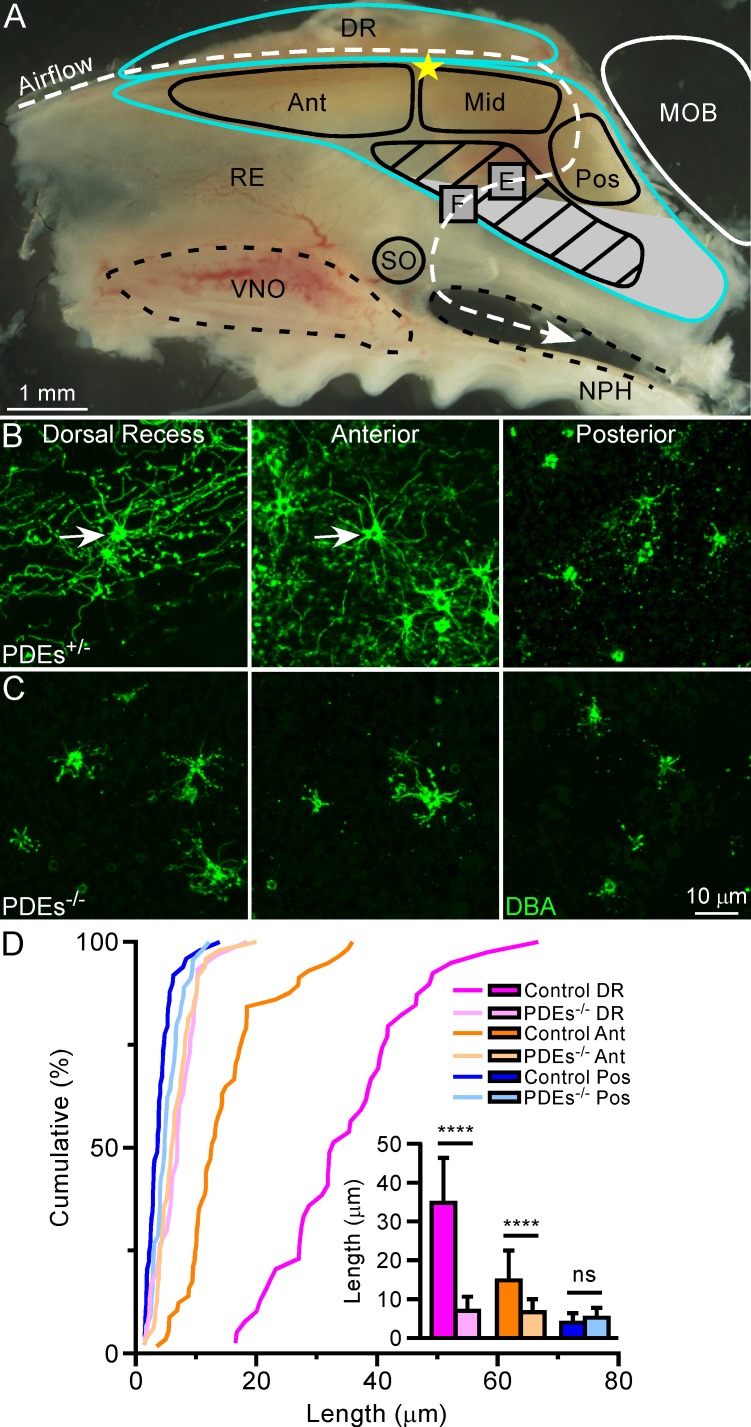
Genetic ablation of PDEs disrupts the cilia pattern. (**A**) Regional subdivision of the mouse nasal septum (modified from [6]). The white dashed line marks the dorsal airstream during inspiration, and the olfactory epithelium is outlined in blue. The dorsal zone is divided into four regions: Ant = anterior; Mid = middle; Pos = posterior; DR = dorsal recess. The Mid region was previously analyzed and only shown for illustrative purposes. The striped region refers to the comet-like region and is described, along with boxes E and F, in **Fig 2**. The gray region approximately marks the ventral zone. MOB = main olfactory bulb; NPH = nasopharynx; RE = respiratory epithelium; SO = septal organ; VNO = vomeronasal organ. The yellow star marks the most dorsal point where the nasal septal cartilage intersects with the ethmoid bone and was used to facilitate comparisons between tissues (see Materials and Methods). (**B** and **C**) Whole-mount olfactory epithelia from PDE1C^+/-^;PDE4A^+/-^ (simplified as PDEs^+/-^) (**B**) and PDEs^-/-^ mice (**C**) were stained with DBA, which labels a subset of OSNs [6, 16]. Confocal images were taken from the dorsal recess (magenta), anterior (orange), and posterior (blue), as shown in (A). Scale bar in (C) applies to all images. Arrows mark dendritic knobs. (**D**) Cumulative frequency (%) of cilia length from DBA^+^ cells in different regions. The bar graph shows quantification of cilia length (mean ± SD); Tukey’s multiple comparisons test, **** p < 0.0001. See also **Table 1**.

In mouse OSNs, decreased cAMP signaling [12] or ablation of ACIII [6] results in shortened cilia, suggesting that cAMP signaling or ACIII may positively regulate olfactory cilia length. Given these findings, we asked whether OSNs with decreased ACIII levels, and presumably cAMP signals, exhibit defects in cilia length. To answer this question, we examined the cilia pattern via the plant lectin *Dolichos biflorus* agglutinin (DBA) [16] staining when two olfactory PDEs, PDE1C and PDE4A, are genetically ablated [11]. PDEs^-/-^ mice have two potential side effects: reduced ACIII levels and prolonged, odor-induced cAMP levels. We previously showed that disruption of odor-induced cAMP signaling through G_olf_ does not alter the cilia pattern [6], suggesting that the major effects of PDE deletion would be due to decreased ACIII expression. Consistent with this notion, we observed much shorter cilia in the dorsal recess and anterior septum of PDEs^-/-^ mice compared to controls (**Fig 1** and **Table 1**). The cilia pattern is therefore significantly disrupted, similar to the phenotype we observed in ACIII^-/-^ mice [6] (**Table 1**). These data support the hypothesis that ACIII level is positively correlated with olfactory cilia length and provide additional evidence that ACIII may be involved in regulating cilia growth and the establishment of the cilia pattern in the dorsal zone.

**Table 1:** Cilia length quantification from mice with altered ACIII levels.

Genotype	Cilia Length (μm, mean ± SD), Cilia Number (n)								Statistical Test	F	p
	DR	n	Ant	n	Pos	n	Comet-like	n			
PDEs^+/-^	34.8 ± 11.6	39	14.8 ± 7.7	57	4.0 ± 2.4	49	**25.7 ± 6.6**	**35**	Two-way ANOVA	125.4 *	< 0.0001
PDEs^-/-^	7.0 ± 3.7	30	6.7 ± 3.4	48	5.2 ± 2.5	45	**6.5 ± 2.3**	**56**			
ACIII^+/+^	*27*.*0 ± 16*.*2*	*39*	*14*.*7 ± 6*.*2*	*48*	*5*.*1 ± 2*.*6*	*43*	**23.4 ± 12.6**	**60**	*Two-way ANOVA*	*27*.9 *	*< 0*.*0001*
ACIII^-/-^	*9*.*3 ± 8*.*5*	*59*	*8*.*6 ± 8*.*1*	*50*	*5*.*5 ± 4*.*6*	*52*	**72.5 ± 23.1**	**37**			
							**One-way ANOVA; F = 202.8; p < 0.0001**				

**Fig 2:**
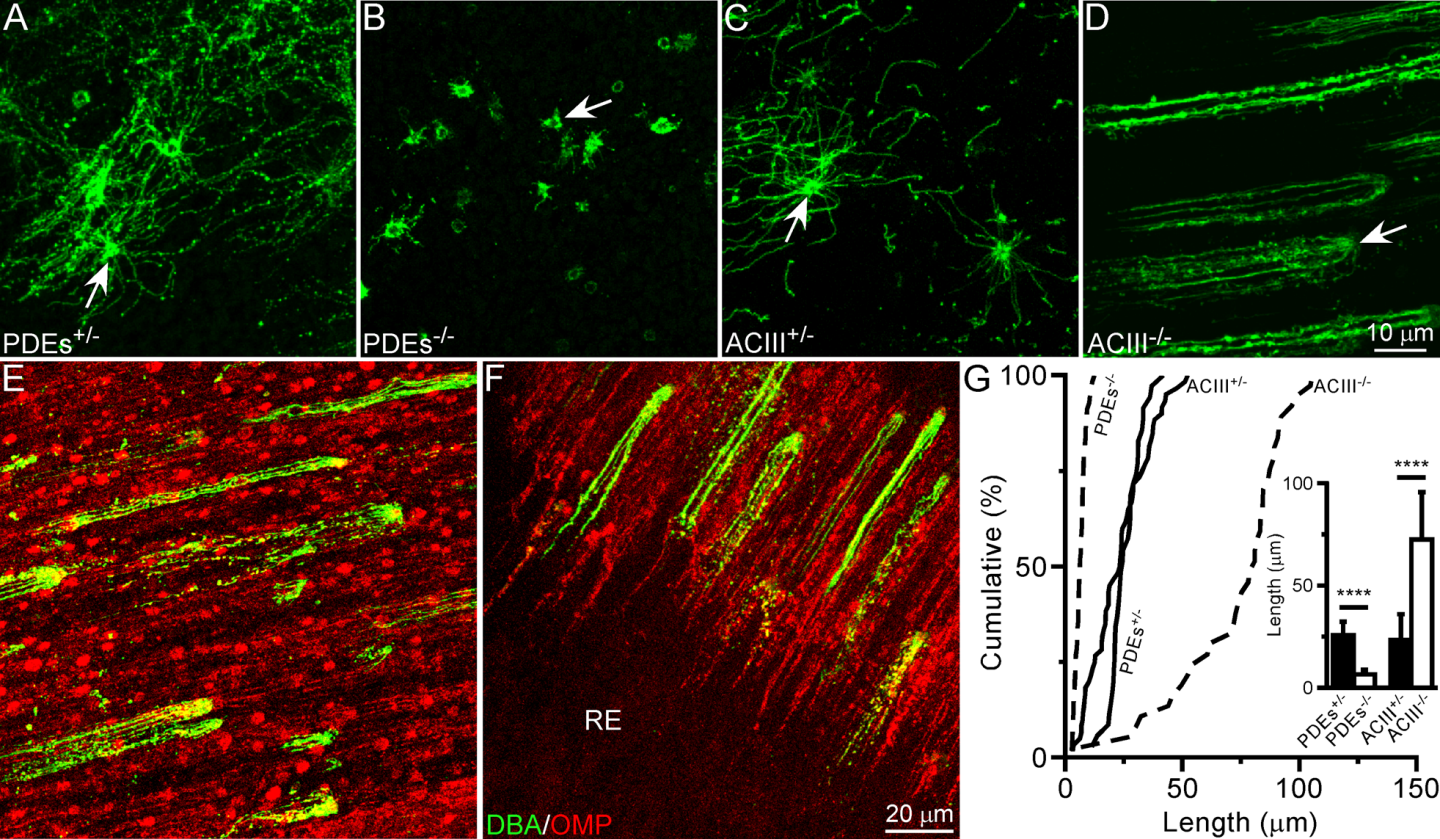
Ablation of PDEs or ACIII differentially impacts cilia length. (**A-D**) Whole-mount olfactory epithelia from PDEs^+/-^ (**A**), PDEs^-/-^ (**B**), ACIII^+/+^ (**C**), and ACIII^-/-^ (**D**) mice were stained with DBA. Images were taken from the comet-like region, as shown in **Fig 1A**. Scale bar in (D) applies to all images. Arrows mark dendritic knobs. (**E** and **F**) DBA^+^ cells with comet-like morphology in ACIII^-/-^ mice appear similar in size and shape to OMP^+^ cells (red). Colocalization is not always obvious, potentially due to variable OMP expression in OSNs. Boxes E and F in **Fig 1A** indicate the approximate locations in which images were taken. Note the sharp contrast between the olfactory epithelium (with comets) and RE (no staining) in (F). (**G**) Cumulative frequency (%) of cilia length from DBA^+^ cells in the comet-like region of all genotypes. The bar graph shows quantification of cilia length (mean ± SD); Tukey’s multiple comparisons test, **** p < 0.0001. See also **Table 1**.

Standard, bold, and italic texts are used to show which data were compared and analyzed. Standard and bold texts indicate data collected in **Figs 1** and **2**, respectively; italic text indicates previously analyzed data [6]. The asterisk (*) denotes a statistical interaction (genotype x region).

### Ablation of PDEs or ACIII differentially impacts cilia length

Although genetic ablation of PDEs or ACIII results in shorter cilia in the dorsal zone, drastically different phenotypes are observed in a region (termed “comet-like” region) situated in the middle to ventral nasal septum outlined in **Fig 1A**. As expected, cilia of PDEs^-/-^ mice are significantly shorter compared to controls (**Fig 2A** and **2B** and **Table 1**) and similar in length to cilia in the dorsal zone of PDEs^-/-^ animals (**Fig 1C** and **Table 1**). Cilia in the same region of ACIII^-/-^ mice [17], however, have a striking appearance. In contrast to controls (**Fig 2C** and **Table 1**), and the dorsal zone of ACIII^-/-^ animals ([6] and **Table 1**), cilia are extremely long, reaching up to 100 μm (**Fig 2D–2G** and **Table 1**). Furthermore, all cilia align in the same direction, presumably the direction of airflow [18, 19], resulting in a comet-like appearance. We confirmed that these comet-like cells are OSNs by double staining with DBA and olfactory marker protein (OMP), a marker for mature OSNs (**Fig 2E** and **2F**). These results reveal that manipulation of ACIII levels causes drastic, location-dependent differences in olfactory cilia architecture.

## Discussion

Here, we provide further evidence that ACIII level is positively correlated with olfactory cilia length in the dorsal zone of the main olfactory epithelium. Furthermore, we demonstrate that genetic ablation of ACIII has dramatic, region-specific effects on olfactory cilia morphology. Together, these data reveal that ACIII level may differentially modulate cilia architecture in the mammalian nose. The results provide novel insight into the role of ACIII in cilia and sensory biology and enhance our current understanding of regional subdivisions in the olfactory epithelium.

Multiple studies suggest that ACIII or cAMP are involved in positively regulating cilia length in various mammalian cell types [6, 12, 20, 21]. We recently demonstrated that in mouse OSNs, genetic ablation of ACIII results in dramatically shorter olfactory cilia, thereby disrupting the cilia pattern in the dorsal zone [6]. Consistent with this finding, the cilia pattern is similarly disrupted in PDEs^-/-^ mice (**Fig 1**), which have significantly reduced ACIII levels [11]. Together, these data support that ACIII level might influence cilia growth and maintenance in the dorsal zone. There are two ways in which ACIII might exert its effects on cilia length. First, ACIII plays a direct role in regulating cilia length. ACIII itself may act as a structural molecule and provide physical support for cilia. Hence, reduced or ablated ACIII levels in PDEs^-/-^ or ACIII^-/-^ mice, respectively, would be expected to impair the structural integrity of cilia. Since ciliary localization of ACIII is not required for olfactory cilia growth [22], ACIII may be able to exert these effects near the base of the cilia in the dendritic knob. Second, ACIII plays an indirect role in regulating cilia length. ACIII levels impact intracellular cAMP concentrations, which in turn could directly or indirectly affect cilia length. This is consistent with the observation that cAMP induces cilia growth in cultured cells [20, 21] and is positively correlated with cilia length in OSNs [12]. The mechanism underlying the relationship between cAMP and cilia length is not fully understood. However, our previous work showed that genetic ablation of ACIII, but not G_olf_, results in significantly shorter cilia in the dorsal zone [6]. Thus, odor-evoked cAMP signaling through G_olf_ may be short-lived and not sufficient to influence cilia length. Odor-independent cAMP signaling, however, may result in sustained basal cAMP levels that impact cilia growth and maintenance. Interestingly, OSNs in PDEs^-/-^ mice exhibit reduced ACIII expression and presumably slower removal of cAMP (thus leading to prolonged response termination) [11], two competing factors in regulating cAMP levels. Given that odor-induced cAMP signaling is transient and not required for maintaining the cilia pattern [6], we predict that odor-evoked cAMP elevation (even though prolonged compared to wild-type animals) does not significantly impact cilia length in PDEs^-/-^ mice. Rather, the basal cAMP levels in these cells, which might be dramatically reduced due to decreased ACIII expression, could affect cilia growth and maintenance. It is feasible that basal cAMP production influences downstream transcription of cilia-related genes, which in turn may regulate cilia length. Future investigations will need to tease apart the direct or indirect roles of ACIII in more detail.

Contrary to these findings that support ACIII as a positive regulator of olfactory cilia growth, there is also evidence suggesting that ACIII negatively regulates mammalian cilia length [23]. In support of this notion, genetic ablation of ACIII has a striking effect on olfactory cilia architecture in the comet-like region (**Figs 1A** and **2**), where cilia are markedly elongated and comet-shaped, as opposed to star-shaped. Curiously, PDEs^-/-^ mice, which have reduced ACIII levels, do not exhibit this phenotype. These results suggest that the role of ACIII in mediating cilia length is more complex than previously anticipated. It is possible that ACIII works in concert with region-specific factors (e.g., structural molecules and/or sensory stimuli, such as airflow) to regulate cilia architecture in different locations. Intriguingly, ACIII is required for detecting both odors [17] and mechanical force [24, 25]. Since the ventral septum experiences lower airflow rates and odorant concentrations than the dorsal zone [18, 19, 26], it is possible that under normal conditions, ACIII acts together with other location-specific molecules to optimize the detection of odors and mechanical force in the comet-like region. When ACIII is absent, the cells may compensate by elongating their cilia parallel to the direction of airflow to increase their chance of encountering sensory stimuli. It is not known whether cells with comet- and star-shaped cilia have unique functional properties. Given that ACIII^-/-^ mice are functionally and behaviorally anosmic [17], it is unlikely that odor-induced responses would differ between OSNs with different cilia morphologies. It is possible, however, that OSNs in ACIII^-/-^ animals retain ligand-independent signaling since AC2 and AC4 are expressed in olfactory cilia [17]. Additional experiments are needed to explore unidentified functions of OSNs in ACIII^-/-^ mice as well as factors that may be involved in establishing cilia architecture in both the comet-like region and dorsal zone.

## Materials and Methods

### Animals

All animals were housed in conventional (non-barrier) animal facilities and were 3–8 weeks old. PDEs^-/-^ mice were generated by crossing PDE1C^+/-^;PDE4A^-/-^ with PDE1C^-/-^;PDE4A^+/-^ mice. Double heterozygous PDE1C^+/-^;PDE4A^+/-^ mice were provided by Haiqing Zhao’s lab [11]. ACIII^-/-^ mice and ACIII^+/+^ littermate controls were bred from heterozygous ACIII^+/-^ mice and provided by Daniel Storm’s lab [17]. All procedures were approved by the Institutional Animal Care and Use Committee of the University of Pennsylvania.

### Immunohistochemistry

Olfactory epithelia were processed as previously reported [6]. Briefly, mice were anesthetized by ketamine/xylazine injection (200 and 20 mg/kg body weight) and then decapitated. After fixation, the nasal septum was dissected out en bloc and incubated with primary antibodies overnight in blocking solution. The primary antibodies include biotinylated *Dolichos biflorus* agglutinin (DBA) (5 mg/mL, 1:300 working dilution; B-1035, Vector Laboratories, Burlingame, CA, USA) and chicken anti-OMP (1:500; kind gift of Dr. Qizhi Gong, University of California, Davis). The septum was washed and incubated for 1–2 h at room temperature with secondary antibodies conjugated to Alexa Fluors (Life Technologies). The secondary antibodies include streptavidin conjugate (1:1000; S32354 and S11226) and goat anti-chicken IgG (1:400; A21449). Olfactory epithelia were marked with a small quantity of tissue dye (1163, Bradley Products, Inc., Bloomington, MN, USA) at a fixed location (i.e., the most dorsal point where the septal cartilage meets the ethmoid bone; see yellow star in **Fig 1A**). Olfactory mucosa were peeled away from the underlying cartilage and bone and mounted in Vectashield mounting medium (Vector Laboratories). Images (z-step = 1 μm) were taken using a Leica TCS SP5 II confocal microscope (Leica Microsystems) with a 40x oil objective. Cilia were traced using Leica LAS AF Lite software, as previously described [6].
